# *Plasmodium falciparum* hydroxymethylbilane synthase does not house any cosynthase activity within the haem biosynthetic pathway

**DOI:** 10.1099/mic.0.001095

**Published:** 2021-10-18

**Authors:** Alan F. Scott, Evelyne Deery, Andrew D. Lawrence, Martin J. Warren

**Affiliations:** ^1^​ School of Biosciences, University of Kent, Canterbury, Kent, CT2 7NJ, UK; ^2^​ Quadram Institute Bioscience, Norwich Research Park, Norwich, NR4 7UQ, UK; ^†^​ Present address: School of Chemistry, Cardiff University, Cardiff, CF10 3AT, UK

**Keywords:** haem synthesis, hydroxymethylbilane, porphobilinogen deaminase, *Plasmodium falciparum*, uroporphyrinogen III

## Abstract

Uroporphyrinogen III, the universal progenitor of macrocyclic, modified tetrapyrroles, is produced from aminolaevulinic acid (ALA) by a conserved pathway involving three enzymes: porphobilinogen synthase (PBGS), hydroxymethylbilane synthase (HmbS) and uroporphyrinogen III synthase (UroS). The gene encoding uroporphyrinogen III synthase has not yet been identified in *Plasmodium falciparum*, but it has been suggested that this activity is housed inside a bifunctional hybroxymethylbilane synthase (HmbS). Additionally, an unknown protein encoded by PF3D7_1247600 has also been predicted to possess UroS activity. In this study it is demonstrated that neither of these proteins possess UroS activity and the real UroS remains to be identified. This was demonstrated by the failure of codon-optimized genes to complement a defined *Escherichia coli hemD* ^−^ mutant (SASZ31) deficient in UroS activity. Furthermore, HPLC analysis of the oxidized reaction product from recombinant, purified *P. falciparum* HmbS showed that only uroporphyrin I could be detected (corresponding to hydroxymethylbilane production). No uroporphyrin III was detected, showing that *P. falciparum* HmbS does not have UroS activity and can only catalyze the formation of hydroxymethylbilane from porphobilinogen.

## Introduction

**Fig. 1.**
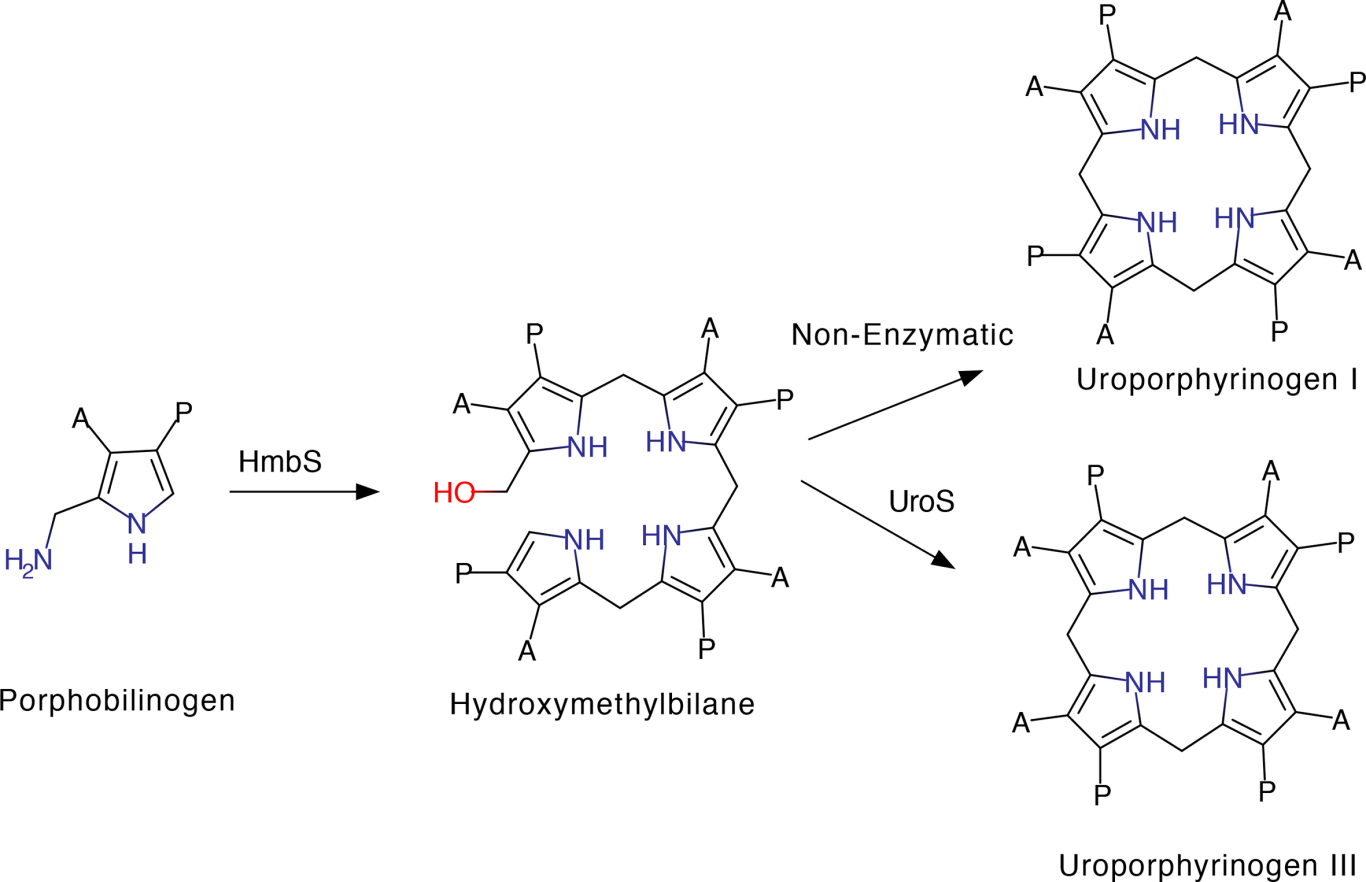
Reactions of HmbS and UroS. HmbS polymerizes porphobilinogen into hydroxymethylbilane, which auto-cyclizes to uroporphyrinogen I. If UroS is present, then hydroxymethylbilane is cyclized into uroporphyrinogen III, a reaction that involves the inversion of ringD. A, acetic acid; *P*, propionic acid.

Haem, as an iron-containing porphyrin, is a modified tetrapyrrole that is derived from the starting material 5-aminolevulinic acid (5-ALA) [1]. The construction of the macrocyclic framework of haem is mediated in just three steps [1]. Firstly, two molecules of 5-ALA are condensed to give a pyrrole, porphobilinogen (PBG), in a reaction catalyzed by PBG synthase [2, 3]. The next step involves the polymerization of four pyrrole units (termed A–D) into a linear bilane called hydroxymethylbilane (HMB) and is mediated by an enzyme called HMB synthase (HmbS) that deaminates and links together four molecules of PBG [4–7]. Finally, the bilane undergoes cyclization, but only after inversion of the terminal D ring, to give uroporphyrinogen III [6, 7]. These three steps are found in all organisms that make modified tetrapyrroles [1]. These reactions are shown in Fig. 1.

A pathway for haem biosynthesis is found in *Plasmodium falciparum*, the protozoan parasite and causative agent of malaria [8]. As a haematophagous organism (blood-feeding parasite), it exists for part of its life cycle in a haem-rich environment and releases large quantities of haem as an insoluble crystalline material called haemozoin [9]. Nonetheless, it requires a functional haem synthesis pathway for survival in the liver and mosquito growth stages [10, 11]. Recent studies have biochemically characterized the complete set of haem synthesis enzymes from *P. falciparum* with the notable exception of uroporphyrinogen III synthase (UroS, formerly HemD) [12–18]. This enzyme is sometimes called uroporphyrinogen III cosynthase, as it often co-purifies with hydroxymethylbilane synthase (HmbS, formerly porphobilinogen deaminase or HemC), and both of these enzymes are required to make uroporphyrinogen III from PBG [5, 19]. HmbS catalyzes the synthesis of an unstable linear tetrapyrrole, HMB [4, 6, 7]. This rapidly cyclizes into uroporphyrinogen I unless the cosynthase is present to invert the terminal ring and cyclize HMB into uroporphyrinogen III [6, 7, 20]. This is the only isomer that can proceed through the haem synthesis pathway. A candidate gene encoding UroS in *P. falciparum* (PF3D7_1247600) has been identified by bioinformatics, but there have been no biochemical studies to validate the finding [21]. Another report in the literature has suggested that the parasite does not need a separate cosynthase because UroS activity can be found within a bi-functional HmbS that houses both HMB synthase and uroporphyrinogen III cosynthase activities [18]. The evidence presented for this was HPLC identification of the (oxidized) reaction product as uroporphyrin III from both native and recombinant HmbS when incubated with PBG.

Although such dual activity has previously been reported for HmbS from *Leptospira interrogans,* this is a very different protein from *P. falciparum* HmbS, being a fusion of HmbS and UroS enzymes [22]. Conversely, the *P. falciparum* HmbS is clearly not a fusion protein because it has similarity to other HmbS enzymes throughout its entire sequence length (with the exception of the N-terminal apicoplast localization sequence, but dual activity was claimed for a truncated HmbS without this signal sequence) [18]. There are some short inserts in the *P. falciparum* sequence, but it is unlikely that UroS activity is contained within these inserts because they are not very long – the longest is 31 amino acids. The sequence does not align to known UroS sequences. A multiple sequence alignment is shown in Figs S1 and S2 (available in the online version of this article). It is, therefore, hard to understand how this enzyme could house two very different activities. Consequently, this report investigates more closely the evidence for dual activity using genetic complementation studies and analytical chemistry. The possibility that UroS activity is encoded by PF3D7_1247600 is also investigated.

## Experimental procedures

### Gene cloning

Synthetic, codon-adapted genes, based on PF3D7_1209600 (*hemC*) and PF3D7_1247600 (putative *hemD*), were purchased from GeneArt for optimal expression in *Escherishia coli* (Fig. S3) and subcloned into a *pET-3a* vector (Novagen) using *NdeI* and *SpeI* restriction sites (the *pET-3a* had been modified to include a *SpeI* site 5′ of the *BamHI* site). Two further constructs were made containing a truncated version of the *hemC* gene to remove a potential signal sequence from the protein product [18]. The truncated gene was obtained by PCR using the following primers: 5′ primer containing *NdeI* site and start codon: CACCATATGGGCATCAAAGATGAAATTATTATCGG; 3′ primer containing *SpeI* site and stop codon: ctcactagttatttattgttcagcagg.

The PCR product was ligated into *pET-3a* and *pET-14b* (Novagen) *usingNdeI* and *SpeI* restriction sites (both vectors had been previously modified to include a *SpeI* site 5′ of the *BamHI* site).

The constructs were sequenced by GATC Biotech to check for the correct insert and reading frame and the absence of mutations.

### Complementation studies

A defined *hemD^−^* mutant SASZ31 (CGSC# 7153 Coli Genetic Stock Center, Yale University) [23] was transformed with the following plasmids: pET-3a empty vector; pET-14b empty vector; pET-3a *hemD P. falciparum* 3D7_1247600; pET-3a *hemC P. falciparum hemC*; pET-3a *hemC^truncated^ P. falciparum hemC^truncated^*; pET-14b *hemD B. megaterium hemD* [24]; pET-14b *hemD E. coli hemD* (a kind gift from Professor Peter Shoolingin-Jordan, Southampton).

The transformations were plated onto LB (lysogeny broth) agar plates with 100 µg ml^−1^ ampicillin and 2 % glucose and incubated at 37 °C for 24 h. They were examined for growth and left for an additional 24 h, after which the colonies were restreaked onto fresh plates. After incubation at 37 °C for 24 h the plates were examined for growth.

### Protein overproduction and purification

BL21^STAR^ (DE3) pLysS (Invitrogen) was transformed with the appropriate construct and a 1 l culture of the resulting strain was grown in LB at 37 °C with shaking to an OD_600_ of 0.6. Gene expression was induced for 20 h at 19 °C by adding 0.4 mM IPTG. Cells were harvested by centrifugation at 4000 r.p.m. for 15 min at 4 °C. The pellet was resuspended in 15 ml resuspension buffer containing 20 mM Tris/HCl pH 8.0, 500 mM NaCl, 5 mM imidazole.

Cells were lysed on an ice-water slurry by sonication at 60 % amplitude for 3 min at 30 s intervals. The lysate was spun for 15 min at 19 000 r.p.m. and the supernatant loaded onto a Ni^2+^–Sepharose column (GE Healthcare) preequilibrated with resuspension buffer. The column was washed with resuspension buffer containing 50 mM imidazole and eluted with resuspension buffer containing 400 mM imidazole. The protein was buffer exchanged with a PD-10 column (GE Healthcare) into 50 mM Tris/HCl pH 8.0, 100 mM NaCl.

### Identification of the reaction product

Purified recombinant HmbS was heated to 60 °C for 10 min on a heat block prior to the assay to deactivate any contaminating UroS. HmbS (25 µg) was incubated with 200 µM porphobilinogen at 37 °C in 0.1 M Tris/HCl pH 8.0. After 1 h, the reaction was stopped by diluting 10× into 1 M HCl. The reaction product was oxidized by adding 10 µl of a 1 mg ml^−1^ benzoquinone in methanol and incubating for 60 min. The mixture was run on an HPLC to identify which uroporphyrin isomer was present. Commercial standards of uroporphyrin I and III (Frontier Scientific) were also run to aid identification.

The uroporphyrin I and III isomers were separated on an ACE 5 AQ column, dimensions 250×4.6 mm, using an Agilent 1100 HPLC system with a flow rate of 1.0 ml min^−1^. The mobile phase was 1 M ammonium acetate pH 5.16 and the organic phase was acetonitrile. A 100 µl sample was injected onto the column (temperature 25 °C) and the porphyrins were detected by their absorbance at 405 nm. A gradient elution was used rising from 13 to 30 % acetonitrile in 25 min and held there for a further 5 min. This was adapted from the protocol used elsewhere [18].

### Enzyme activity assay

Enzyme at various concentrations was incubated with 100 µM porphobilinogen at 37 °C in 0.1 M Tris/HCl pH 8.0. After 25 min, the reaction was stopped by diluting 10× into 1 M HCl. The reaction product was oxidized by adding 10 µl of a 1 mg ml^−1^ benzoquinone in methanol and incubating for 60 min. Absorbance was read at 405 nm and the amount of uroporphyrin calculated using the extinction coefficient of 54.8×105 M^−1^ l.

## Results

### Complementation studies

To test if either *P. falciparum hemC* (encoding HmbS) or *P. falciparum* 3D7_1247600 (putative coding sequence for UroS) harbour UroS activity, complementation studies were performed to see if either gene could restore growth to a defined *hemD^−^* mutant (SASZ31) lacking UroS activity [23]. Two *P. falciparum hemC* constructs were used, both of which were codon-optimized for expression in *E. coli *. One contained the full-length *hemC* gene in a *pET-3a* vector and the other a truncated *hemC* gene, also in a *pET-3a* vector. The truncation removed a signal sequence known to hinder gene expression in *E. coli* and has been shown not to be essential for activity [18].

The *hemD^−^* mutant SASZ31 was transformed with these constructs and with control plasmids. The controls included an empty *pET-3a* as a negative control and plasmids harbouring known *hemD* genes from *Bacillus megaterium* and *E. coli* as positive controls. As these control genes were in a *pET-14b* plasmid, an empty *pET-14b* was also used as a further control.

The resulting strains were grown on LB agar at 37 °C and the size of colonies was noted at 24 and 48 h. To test for the viability of the colonies after 48 h they were restreaked onto a fresh LB agar plate and incubated at 37 °C for 24 h. The plates were examined for colonies.

The control plasmids harbouring known *hemD* genes were able to restore normal growth to the *hemD^−^* mutant. However, the empty vectors and both the *P. falciparum hemC* constructs and the *P. falciparum* 3D7_1247600 construct were unable to restore normal growth. This demonstrates that neither the *P. falciparum hemC* gene nor *P. falciparum* 3D7_1247600 can complement an *E. coli hemD^−^* mutant, showing that neither encodes for UroS activity. The results are shown in Table 1.

**Table 1.** Attempts to complement a *hemD^−^* mutant with *P. falciparum hemC*

Construct	24 h growth	48 h growth	Restreaked 24 h growth
pET-3a	+	++	−
pET-14b	+	++	+
pET-3a *hemC* (*P. falciparum*)	+	++	−
pET-3a *hemC^truncated^* (*P. falciparum*)	+	++	−
pET-3a *hemD* (*P. falciparum*)	+	++	+
pET-14b *hemD* (* E. coli *)	++++	++++	++++
pET-14b *hemD* (* B. megaterium *)	++++	++++	++++

*hemD* ^−^ mutant SASZ31 was transformed with various constructs and incubated on LB agar plates with 0.2 % glucose and appropriate antibiotics at 37 °C for 48 h. The size of any resultant colonies was recorded after 24 and 48 h. To test for viability, the colonies were restreaked onto a fresh plate and grown for a further 24 h and examined for evidence of growth. The growth is indicated in the table by the number of plus signs from + (poor growth) to ++++ (normal growth). A indicates that no growth was observed.

### Protein overproduction in *E. coli* and identification of the reaction product-

A *pET-14b* construct harbouring the *P. falciparum hemC* gene in frame with an N-terminal hexa-His tag coding sequence was used for protein production in *E. coli *. The *hemC* gene was codon-optimized for *E. coli* and lacked the apicoplast localization sequence. The overproduced protein was mostly insoluble but a small quantity of soluble protein was successfully purified to homogeneity from the cell lysate using Ni^2+^ affinity chromatography. The purity was assessed by SDS-PAGE (Fig. S4).

**Fig. 2.**
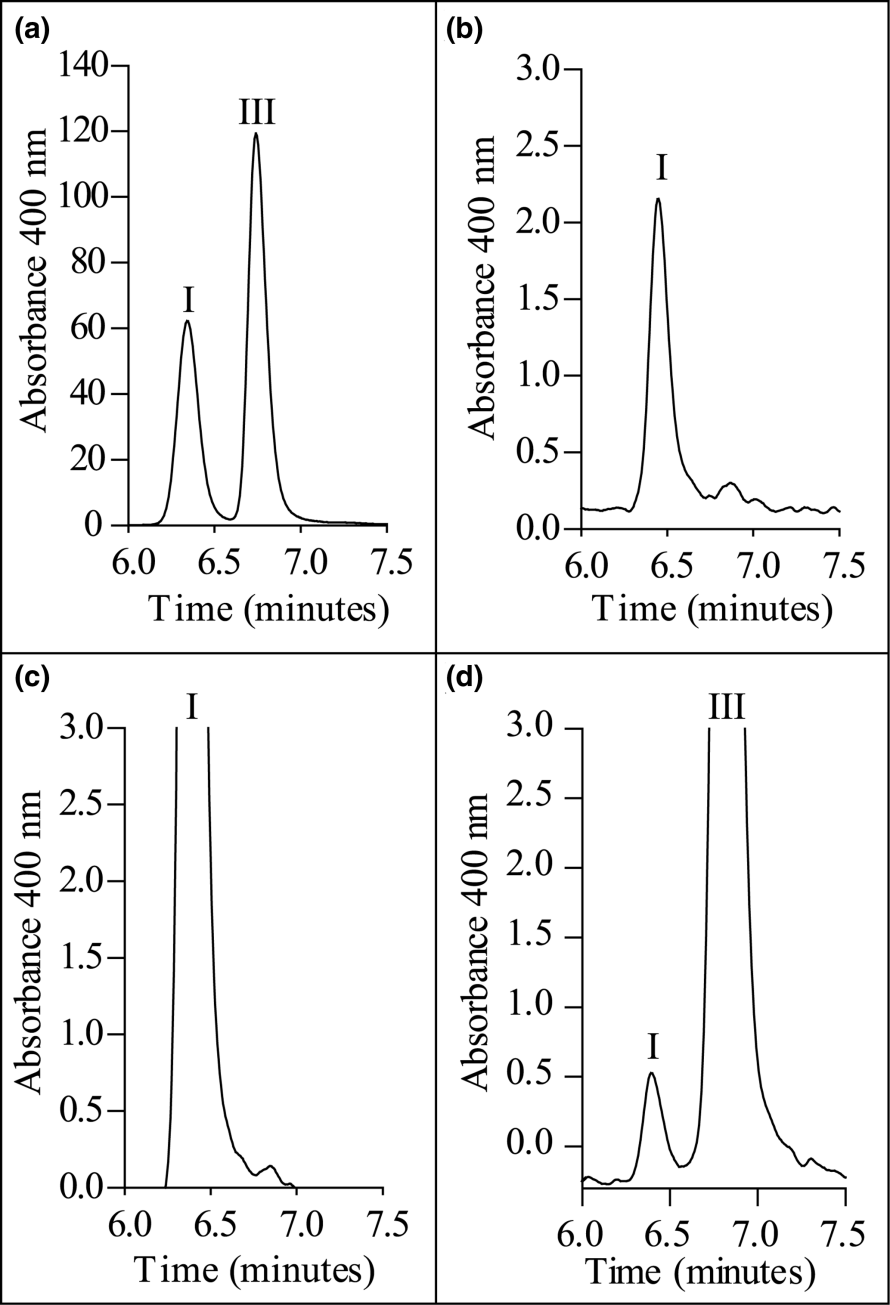
HPLC analysis of HmbS reaction product. HPLC traces showing (**a**) commercial standards of uroporphyrin I (left) and III (right) and (**b**) the oxidized reaction product of HmbS alone and (**c**) spiked with uroporphyrin I and (**d**) uroporphyrin III.

The purified protein was subjected to a 60 °C heat treatment for 10 min to deactivate any contaminating UroS. *P. falciparum* HmbS was demonstrated to be resistant to heat treatment in the original study [18]. The protein was incubated with substrate for 60 min at 37 °C and the resulting product was oxidized with HCl and benzoquinone. This sample was analysed by HPLC to see if the product was uroporphyrin I (corresponding to hydroxymethylbilane) or uroporphyrin III (corresponding to uroporphyrinogen III). Identification was by comparison with commercial standards of uroporphyrin I and III. The reaction product matched the retention time of uroporphyrin I. The results are shown in Fig. 2. As a positive control, analysis of known HmbS and UroS enzymes was also performed (Fig. S5). To confirm that the observed reaction product was enzymatically generated by HmbS, the assay was repeated with different concentrations of enzyme and the reaction product was quantified by absorbance spectrometry. A linear correlation was observed between enzyme concentration and the amount of product formed, which is indicative of enzymatic reactions. The results are presented in Fig. S6.

## Discussion

The claim that *P. falciparum* HmbS has UroS activity [18] has been challenged through complementation studies with a *hemD^−^* mutant and HPLC analysis of the reaction product from recombinant enzyme. SASZ31 is a defined *hemD^−^* mutant that grows very poorly [23]. Complementation with control *hemD* genes from *B. megaterium* and *E. coli* was able to restore normal growth to the mutant, but *P. falciparum hemC* could not restore normal growth. Because HmbS has an apicoplast localization sequence that hinders expression but is not required for alleged dual activity [18], a truncated gene lacking this sequence was also made. This also failed to complement the mutant.

Furthermore, the truncated HmbS was overproduced in *E. coli* with an N-terminal hexa-His tag and purified. After incubation with substrate for an hour at 37 °C, the sample was oxidized and run on HPLC along with commercial standards of uroporphyrin I and uroporphyrin III. The HPLC result clearly identified the enzyme’s oxidized product as uroporphyrin I. No uroporphyrin III could be detected. These results contradict those previously published [18] where HPLC analysis of the reaction product from native and recombinant HmbS identified the (oxidized) reaction product as uroporphyrin III. This conflict could be explained by the presence of a contaminating UroS in the earlier study. Although the researchers used heat treatment to denature any UroS (HmbS is heat stable but UroS is not), it is possible that any UroS could have refolded and reactivated itself during the 12 h incubation of heat-treated HmbS with substrate [25–27]. Further, the assay buffer contained additives known to increase the stability of UroS [27].

Our results clearly demonstrate that the previous claim that *P. falciparum* HmbS contains uroporphyrinogen III synthase (UroS) activity is mistaken [18]. Another report [21] has postulated that UroS activity could reside in the protein encoded by PF3D7_1247600. Our complementation studies have shown that this is also incorrect. It should now be a matter of importance to find the gene that encodes for the real uroporphyrinogen III synthase.

## Supplementary Data

Supplementary material 1
