# Supplementary material for: Plasmodium falciparum hydroxymethylbilane synthase does not house any cosynthase activity within the haem biosynthetic pathway
Source: Microbiology (Reading). 2021 Oct 18;167(10):001095. doi: 10.1099/mic.0.001095 (PMC8698207; doi:10.1099/mic.0.001095)
Supplement: Supplementary material 1 [file mic-167-1095-s001.pdf]

Supporting Information for:

Plasmodium falciparum hydroxymethylbilane synthase does not house any cosynthase activity within the haem biosynthetic pathway

Alan F. Scott, Evelyne Deery, Andrew D. Lawrence, Martin J. Warren

Contents:

S1. Multiple sequence alignment for *P. falciparum* HmbS

S2. Multiple sequence alignment for *P. falciparum* HmbS and HemD

S3. Sequence of synthetic *hemC* gene encoding *P. falciparum* HmbS

S4. SDS-PAGE showing purification of recombinant *P. falciparum* HmbS

S5. HPLC analysis of oxidised reaction products

S6. Amount of uroporphyrin I produced by *P. falciparum* HmbS

```
human          1   ......................................................MSGNGNAAATAEENSP
E_coli         1   ......................................................MLDN............
B_megaterium   1   ......................................................MRK.............
A_thaliana     1   MDIASSSLSQAHKVVLTRQPSSRVNTCSLGSVSAIGFSLPQISSPALGKCRRKQSSSGFVKACVAVEQKT
P_falciparum   1   ....MHLLSFLSFIIWFIHCTAKRHEYSIKKYFLNSHNFCKIKPDPFRKDTLKKRLYS.........SDG

human         17   KMRVIRVGTRKSQLARIQTDSVVATLKASYPGLQFE....IIAMSTTGDKILD.TALSKIGEKSLFTKEL
E_coli         5   ...VLRIATRQSPLALWQAHYVKDKLMASHPGLVVE....LVPMVTRGDVILD.TPLAKVGGKGLFVKEL
B_megaterium   4   ....IIVGSRRSKLALTQTKWVIEQLKKQGLPFEFE....IKEMVTKGDQILN.VTLSKVGGKGLFVKEI
A_thaliana    71   RTAIIRIGTRGSPLALAQAYETREKLKKKHPELVEDGAIHIEIIKTTGDKILS.QPLADIGGKGLFTKEI
P_falciparum  58   IKDEIIIGTRDSPLALKQSEKVRKKIMSYFKKMNKNINVTFKYIKTTGDNILDSKSVGLYGGKGIFTKEL

human         82   EHALEKNEVDLVVHSLKDLPTVLPPGFTIGAICKRENPHDAVVFHPKFVGKTLETLPEK...........
E_coli        67   EVALLENRADIAVHSMKDVPVEFPQGLGLVTICEREDPRDAFVSN...NYDSLDALPAG...........
B_megaterium  65   EQAMLDKEIDMAVHSMKDMPAVLPEGLTIGCIPLREDHRDALISK...NGERFEELPSG...........
A_thaliana   140   DEALINGHIDIAVHSMKDVPTYLPEKTILPCNLPREDVRDAFICL...TAATLAELPAG...........
P_falciparum 128   DEQLINGNVDLCVHSLKDVPILLPNNIELSCFLKRDTINDAFLSIKYKSINDMNTVKSVSKTEDIHHINK

human        141   ..........SVVGTSSLRRAAQLQRKFPHLEFRS.IRGNLNTRLRKLDEQQEFSAIILATAGLQRMGWH
E_coli       123   ..........SIVGTSSLRRQCQLAERRPDLIIRS.LRGNVGTRLSKLD.NGEYDAIILAVAGLKRLGLE
B_megaterium 121   ..........AVIGTSSLRRGAQLLSMRSDIEIKW.IRGNIDTRLEKLK.NEDYDAIILAAAGLSRMGWS
A_thaliana   196   ..........SVVGTASLRRKSQILHKYPALHVEENFRGNVQTRLSKLQ.GGKVQATLLALAGLKRLSMT
P_falciparum 198   KDSDHNNDTLCTIGTSSLRRRSQIKNRYKNIYVNN.IRGNINTRIEKLY.NGEVDALIIAMCGIERLIKK

human        200   NR................................VGQILHPEECMYAVGQGALGVEVRAKDQDILDLVGV
E_coli       181   SR................................IRAALPPEISLPAVGQGAVGIECRLDDSRTRELLAA
B_megaterium 179   KDT...............................VTQYLEPEISVPAVGQGALAIECRENDHELLSLLQA
A_thaliana   255   EN................................VASILSLDEMLPAVAQGAIGIACRTDDDKMATYLAS
P_falciparum 266   ANLKHLLKNKEQKNICQPFLLKCNNKKCIDLCHVNIQKLNKNLIYPALGQGIIAVTSHKKNYFISSLLKN

human        238   LHDPETLLRCIAERAFLRHLEGGCSVPVAVHTAMKDG.QLYLTGGVWSLDGSDSIQETMQATIHVPAQHE
E_coli       219   LNHHETALRVTAERAMNTRLEGGCQVPIGSYAELIDG.EIWLRALVGAPDGS..................
B_megaterium 218   LNHDETARAVRAERVFLKEMEGGCQVPIAGYGRILDGGNIELTSLVASPDGK..................
A_thaliana   293   LNHEETRLAISCERAFLETLDGSCRTPIAGYASKDEEGNCIFRGLVASPDGT..................
P_falciparum 336   INNKKSEMMAQIERSFLYHIDGNCMMPIGGYTNMRNE.DIYLHVIINDIHGY..................

human        307   DGPEDDPQLVGITARNIPRGPQLAAQNLGISLANLLLSKGAKNILDVARQLNDAH.....
E_coli       270   .......QIIRGERRGAPQ....DAEQMGISLAEELLNNGAREILAEVYNGDAPA.....
B_megaterium 270   .......TIYKEHITGK......DPIAIGSEAAERLTSQGAKLLIDRVKEELDK......
A_thaliana   345   .......KVLETSRKGPYVY..EDMVKMGKDAGQELLSRAGPGFFGN.............
P_falciparum 387   .......NKYQVTQKDTLY....NYKEIGPNAAIKMKEIIGTEQFNKIKAEAELHLLNNK
```

FIGURE S1 – Multiple sequence alignment for HmbS

This alignment was produced using ClustalW (http://www.ebi.ac.uk/Tools/msa/clustalw2/) and formatted with ESPript (http://espript.ibcp.fr/ESPript/ESPript/). The HmbS sequences come from Human, *Bacillus megaterium*, *Escherichia coli*, *Arabidopsis thalinana* and Pl *Plasmodium falciparum.*

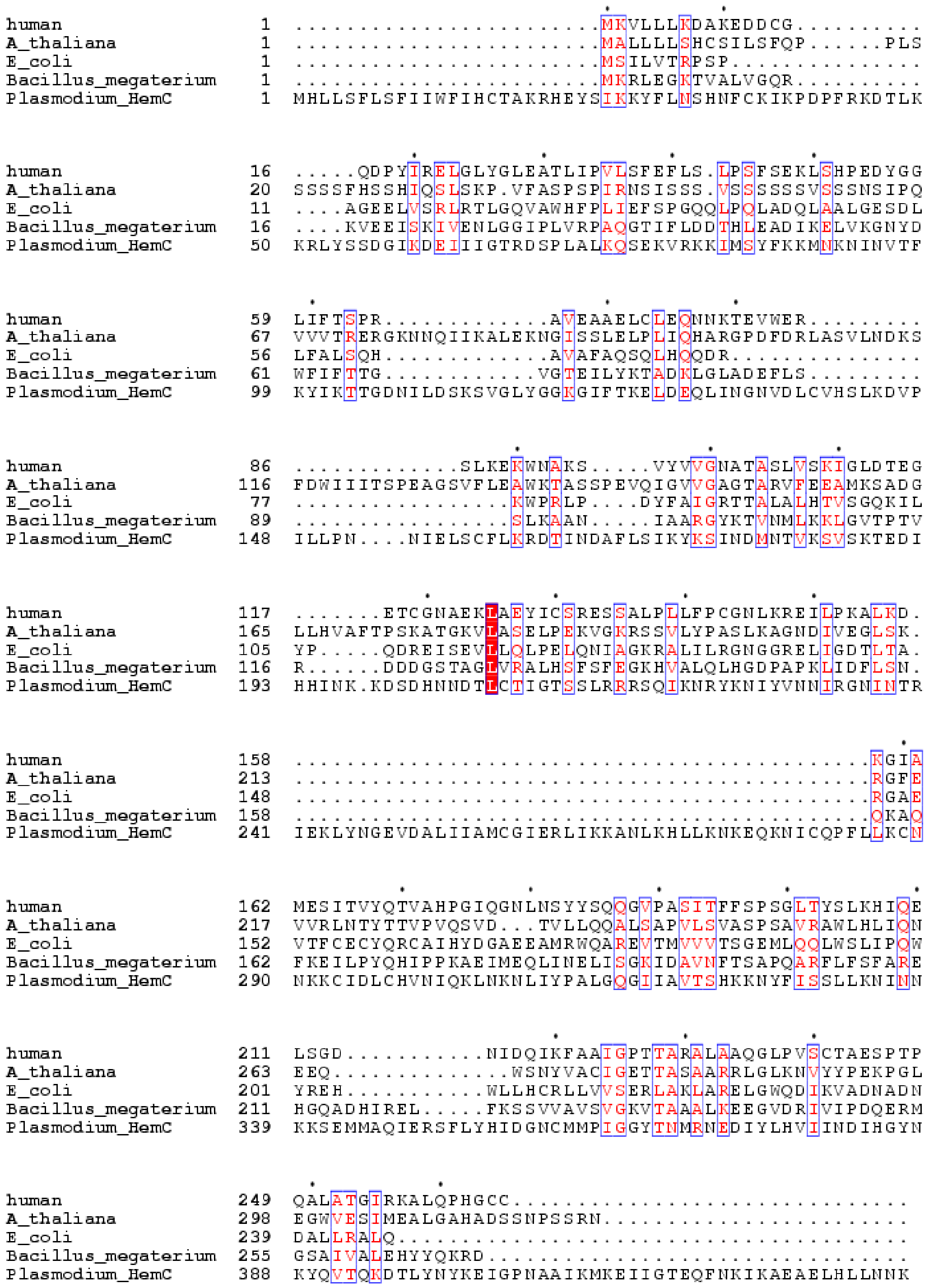

Figure S2- Multiple Sequence Alignment for UroS

This alignment was produced using ClustalW (http://www.ebi.ac.uk/Tools/msa/clustalw2/) and formatted with ESPript (http://espript.ibcp.fr/ESPript/ESPript/). The UroD sequences come from Human, *Bacillus megaterium*, *E. coli*, *Arabidopsis thalinana* and *Plasmodium falciparum* (HmbS).

ATGGGCATCAAAGATGAAATTATTATCGGCACCCGTGATAGCCCGCTGGCCCTGAAACAGAGCGAA
AAAGTGCGCAAAAAAATCATGAGCTACTTCAAAAAAATGAACAAAAACATCAACGTGACCTTCAAAT
ACATTAAAACCACCGGCGATAACATTCTGGATAGCAAAAGCGTGGGCCTGTATGGCGGCAAAGGCA
TTTTTACCAAAGAACTGGATGAACAGCTGATTAACGGCAACGTGGATCTGTGCGTGCATAGCCTGAA
AGATGTGCCGATTCTGCTGCCGAACAACATTGAACTGAGCTGCTTTCTGAAACGTGATACCATCAAC
GATGCGTTTCTGAGCATCAAATATAAAAGCATCAACGATATGAACACCGTGAAAAGCGTGAGCAAA
ACCGAAGATATCCACCACATCAATAAAAAAGATAGCGATCACAACAACGATACCCTGTGCACCATTG
GCACCAGCAGCCTGCGTCGTCGTAGCCAGATTAAAAACCGCTATAAAAACATCTATGTGAACAACAT
TCGCGGCAACATTAACACCCGTATCGAAAAACTGTATAACGGCGAAGTGGATGCGCTGATTATTGCC
ATGTGCGGCATTGAACGCCTGATTAAAAAAGCGAACCTGAAACACCTGCTGAAAAACAAAGAACAG
AAAAACATCTGCCAGCCGTTTCTGCTGAAATGCAACAACAAAAAATGCATCGATCTGTGCCATGTGA
ACATTCAGAAACTGAACAAAAACCTGATTTATCCGGCGCTGGGCCAGGGCATTATTGCGGTGACCAG
CCACAAAAAAAACTACTTCATCAGCAGCCTGCTGAAAAATATTAACAACAAAAAAAGCGAAATGATG
GCGCAGATTGAACGTAGCTTTCTGTATCACATTGATGGCAACTGCATGATGCCGATTGGCGGCTATA
CCAACATGCGTAACGAAGATATCTATCTGCACGTGATTATCAACGATATCCACGGCTACAATAAATAT
CAGGTGACCCAGAAAGATACCCTGTATAACTATAAAGAAATTGGCCCGAACGCGGCGATTAAAATG
AAAGAAATCATCGGCACCGAACAGTTCAACAAAATTAAAGCGGAAGCGGAACTGCACCTGCTGAAC
AATAAA**TAA**

FIGURE S3 – Sequence of synthetic *hemC* gene encoding *P. falciparum* HmbS

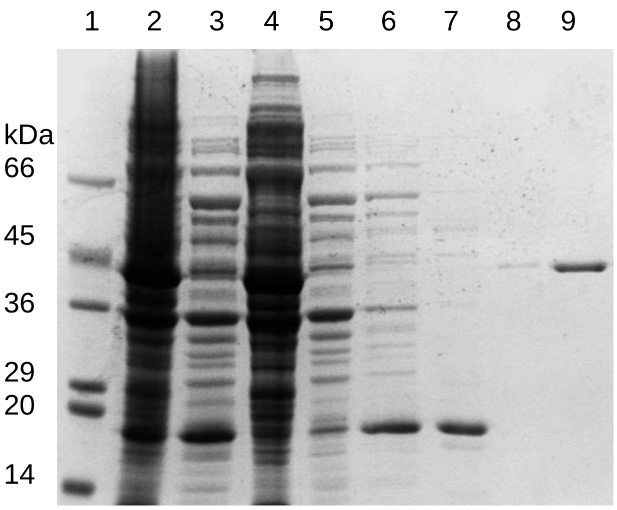

FIGURE S4 – SDS-PAGE showing purification of recombinant *P. falciparum* HmbS

(1) Markers SDS7 (Sigma); (2) Total protein from lysate; (3) Total soluble protein from lysate; (4) Total insoluble protein from lysate; (5) Flow through from Ni-sepharose column; (6) Flow through from 5 mM imidazole wash; (7) Flow through from 50 mM imidazole wash; (8-9) Elution fractions from 400 mM imidazole wash

Expected mass of HmbS: 43.6 kDa

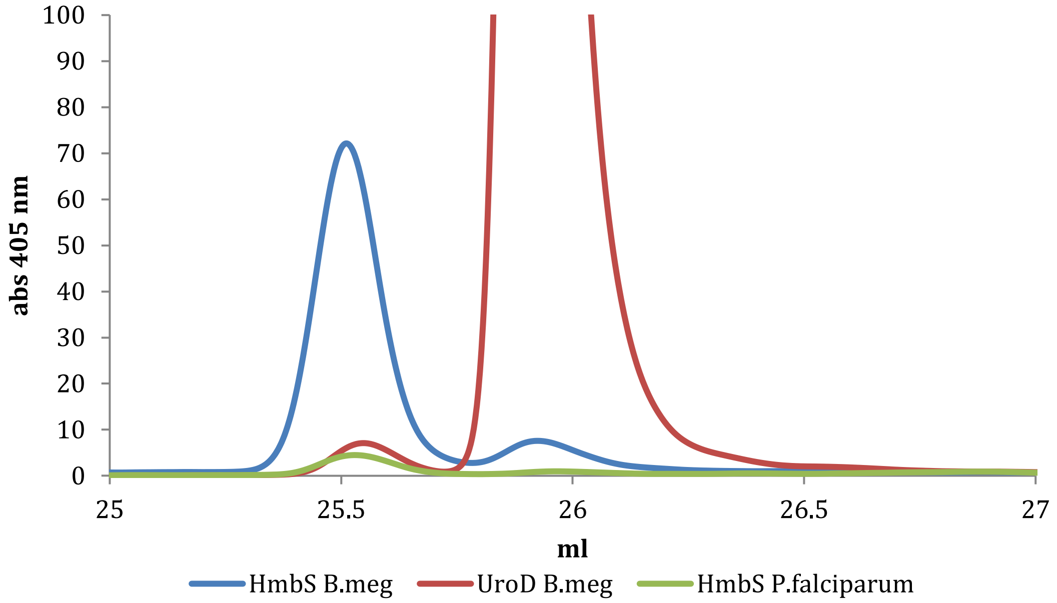

FIGURE S5 – HPLC analysis of oxidised reaction products

Green: oxidised reaction product of *B. meg* HmbS

Purple: oxidised reaction product of *B. meg* HmbS + *B. meg* UroD

Orange: oxidised reaction product of *P. falciparum* HmbS

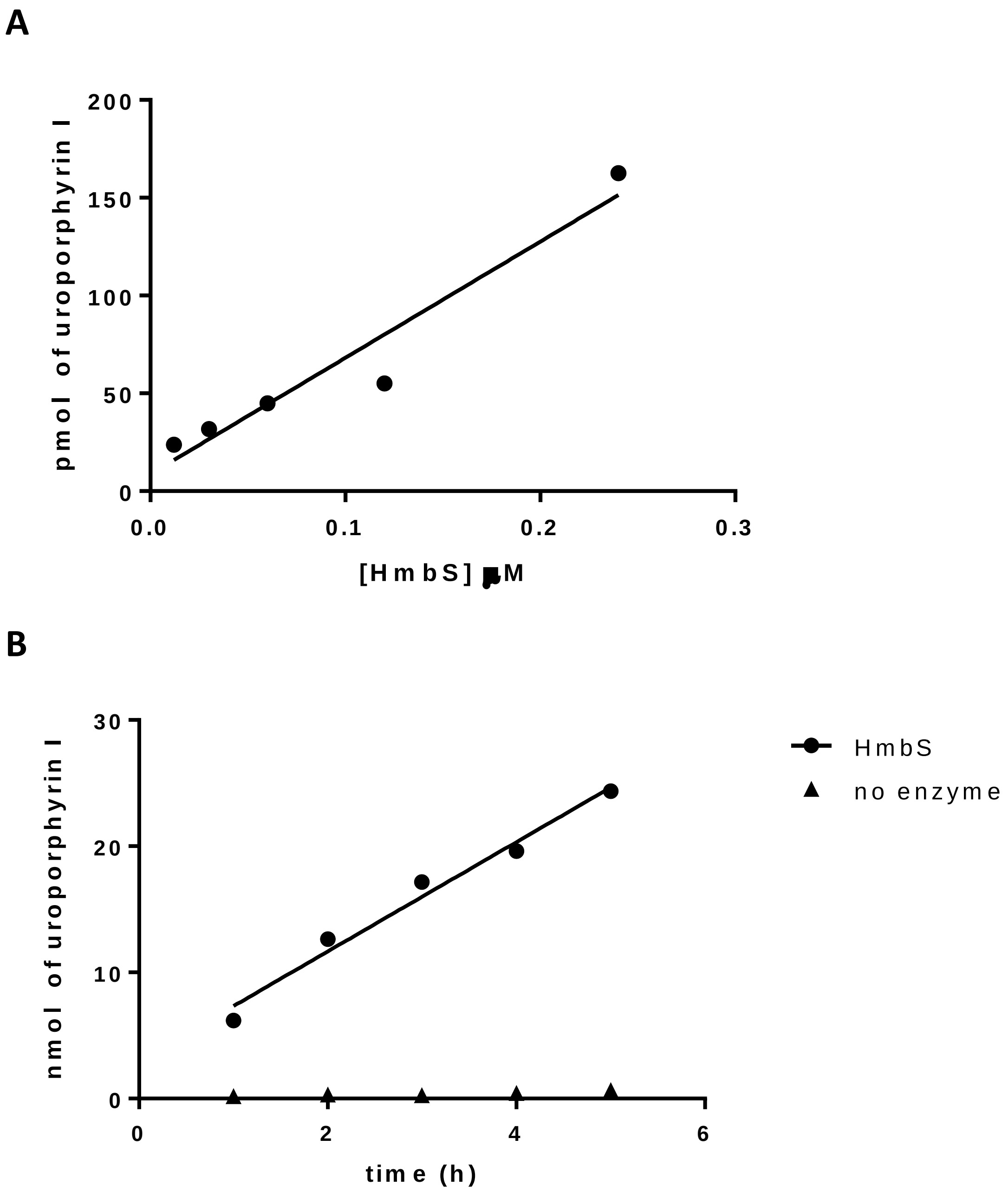

FIGURE S6 – Amount of uroporphyrin I produced by *P. falciparum* HmbS

A) Enzyme was incubated with 100 µM porphobilinogen at 37 °C in 0.1 M Tris HCl pH 8.0. After 25 minutes, the reaction was stopped by diluting 10x into 1 M HCl. The reaction product was oxidised by adding 10 µl of a 1 mg/ml benzoquinone in methanol and incubating for 60 minutes. Absorbance was read at 405 nm and the amount of uroporphyrin calculated using the extinction coefficient e 54.8 $x10^{5}$ M^{-1} L.

B) Enzyme (0.9 mM) was incubated with 200 µM porphobilinogen at 37 °C in 0.1 M Tris HCl pH 8.0. After 60 minutes, an aliquot was stopped by diluting 10x into 1 M HCl. The reaction product was oxidised by adding 10 µl of a 1 mg/ml benzoquinone in methanol and incubating for 60 minutes. Absorbance was read at 405 nm and the amount of uroporphyrin calculated using the extinction coefficient ε 54.8 $x10^{5}$ M^{-1} L.
